# Tumoral Calcium Pyrophosphate Deposition in the Glenohumeral Joint: A Case Report

**DOI:** 10.1155/cro/9540064

**Published:** 2026-11-03

**Authors:** Chikara Watanabe, Yohei Harada, Shuhei Matsumura, Shin Yokoya, Nobuo Adachi

**Affiliations:** ^1^ Department of Orthopaedic Surgery Graduate School of Biomedical and Health Sciences Hiroshima University Hiroshima Japan hiroshima-u.ac.jp; ^2^ Department of Orthopaedic Surgery Hiroshima Citizens Hospital Hiroshima Japan city-hosp.naka.hiroshima.jp

**Keywords:** calcium pyrophosphate deposition disease, case report, glenohumeral joint, tophaceous pseudogout, tumor-like lesion

## Abstract

**Case:**

A 75‐year‐old male patient presented with long‐standing pain and swelling in the shoulder. Radiographic evaluations revealed a large, calcified mass posterior to the humeral head, without apparent bone erosion or destruction. Magnetic resonance imaging demonstrated an intra‐articular mass‐like lesion in the glenohumeral joint without apparent extension into the adjacent bone or surrounding soft tissues. Because the lesion appeared nonaggressive on imaging, a benign calcified intra‐articular lesion was initially considered. Open and arthroscopy‐assisted excision of the lesion was performed because of severe pain and restricted range of motion. Pathological examination demonstrated abundant crystalline deposits with angular and elongated morphology, consistent with calcium pyrophosphate deposition disease, leading to the diagnosis of tophaceous pseudogout. Two years after the surgery, radiography showed mild enlargement of the residual calcific deposits, although the patient remained asymptomatic.

**Conclusion:**

Tophaceous pseudogout of the glenohumeral joint is rare and may mimic neoplastic lesions on imaging; therefore, it should be considered in the differential diagnosis of calcified masses in the shoulder.

## 1. Introduction

Pseudogout is characterized by the deposition of calcium pyrophosphate dihydrate (CPPD) crystals within joints, leading to acute arthritis following crystal shedding. CPPD crystals rarely form neoplastic or lumpy deposits; however, when they do, the condition is referred to as tophaceous pseudogout. Although there have been reports of tophaceous pseudogout, most cases have been observed in the temporomandibular joint, with a few cases in the hand and knee joints [1, 2]. Cases involving the glenohumeral joint are rare [3]. Here, we present a case of tophaceous pseudogout of the glenohumeral joint. This condition may mimic neoplastic lesions on imaging, making preoperative diagnosis challenging.

The Institutional Review Board (IRB) of Hiroshima University does not require IRB approval for a case report. The patient was informed that data concerning the case would be submitted for publication, and he provided consent.

## 2. Case Report

A 75‐year‐old male presented with pain and swelling in his left shoulder. He had been aware of the pain for over 10 years, which had gradually worsened over the past 2 years, making it difficult to elevate his arm. His medical history included hypertension, urolithiasis, thyroid dysfunction, fulminant hepatitis, and untreated diabetes mellitus. Approximately 3 years before surgery, he had experienced an episode of acute pain in the contralateral shoulder accompanied by fever and marked restriction of shoulder motion for approximately 1 month; however, CPPD was not diagnosed at that time. No documented history of hyperparathyroidism, hemochromatosis, or hypomagnesemia was identified in the available medical records, although specific metabolic investigations for secondary CPPD were not available. Additionally, there was no documented history of CPPD or chondrocalcinosis involving other joints. Physical examination revealed swelling and local heat in the left shoulder, with a limited range of motion: 60° in flexion, 30° in external rotation at the side, and from the hand to the buttock during internal rotation. The results of subacromial impingement tests were negative. Radiography and computed tomography (CT) revealed a calcified mass measuring 41 × 24 mm posterior to the humeral head (Figures 1 and 2). Magnetic resonance imaging (MRI) demonstrated an intra‐articular mass in the glenohumeral joint, showing low signal intensity on T1‐weighted images and heterogeneous low‐to‐high signal intensity on T2‐weighted images. No apparent bone erosion, bone destruction, or extension into the surrounding soft tissues was observed (Figure 3). MRI also demonstrated findings suspicious for a partial‐thickness tear of the supraspinatus tendon. Blood tests showed normal white blood cell count, CRP, and uric acid levels. The Constant–Murley score was 17, the UCLA score was 10, and the pain numerical rating scale (NRS) score was 6.

**Figure 1:**
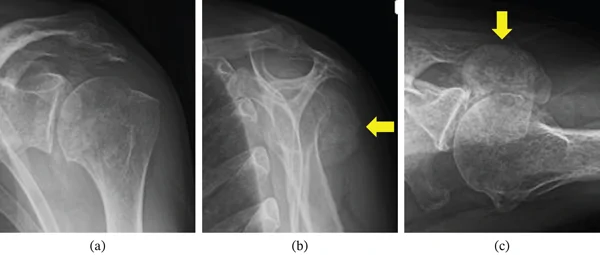
Preoperative radiographs of the glenohumeral joint. The calcified lesion is indicated by an arrow. (a) Anteroposterior view. (b) Scapular Y view. (c) Axillary view of the glenohumeral joint.

**Figure 2:**
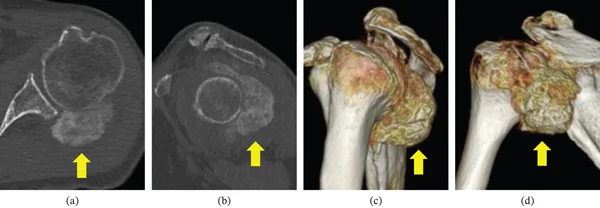
Preoperative computed tomography of the shoulder. The calcified lesion is indicated by the arrow. (a) Axial view. (b) Sagittal view. (c,d) Three‐dimensional images.

**Figure 3:**
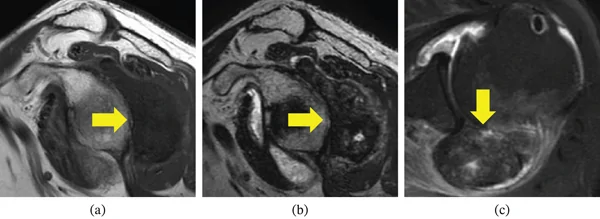
Preoperative magnetic resonance images of the left shoulder. The lesion is indicated by the arrow. (a) T1‐weighted sagittal image. (b) T2‐weighted sagittal image. (c) T2‐weighted fat suppressed axial image.

Because the lesion showed no aggressive imaging features, such as bone destruction or extension into the surrounding soft tissues, a benign calcified intra‐articular lesion was initially considered. Although synovial chondromatosis, hemangioma, and synovial sarcoma were considered in the differential diagnosis, a specific pathological diagnosis could not be established preoperatively. Because the patient had severe pain and marked restriction of shoulder motion, surgical excision was performed. Arthroscopy revealed a soft mass extending from the superior to the posterior aspect of the glenohumeral joint, together with a partial‐thickness tear of the supraspinatus tendon (Figure 4). Because complete arthroscopic removal was difficult owing to the size and posterior location of the lesion, open excision through a posterior approach was performed (Figure 5). The lesion was yellowish‐white, soft, and chalky, and was partially adherent to the joint capsule. The main mass was resected en bloc, followed by arthroscopic removal of the remaining accessible deposits. The partial supraspinatus tear was left untreated because there were no clinical signs of subacromial impingement.

**Figure 4:**
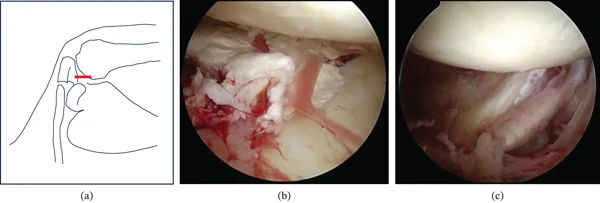
(a) Schema of skin incision, right lateral decubitus position. Arthroscopy was performed through anterior portal. (b) Arthroscopic findings of the lesion. (c) Partial tear of supraspinatus tendon.

**Figure 5:**
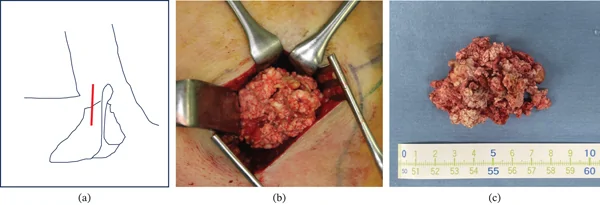
(a) Schema of skin incision from posterior approach. (b) Exposure of the lesion after deltoid and infraspinatus muscle split and incision of the posterior joint capsule. (c) The resected lesion.

Pathological examination revealed no evidence of malignancy but demonstrated abundant deposits of angular and elongated microcrystals, morphologically consistent with CPPD, leading to a diagnosis of tophaceous pseudogout (Figure 6). Radiography and CT on the second postoperative day revealed residual calcium deposits within the supraspinatus tendon (Figures 7 and 8). Postoperatively, pain at rest and during the night significantly improved, along with gradual recovery of range of motion. Two years postoperatively, the radiograph showed mild enlargement of the residual calcific lesion (Figure 9). However, the patient was symptom‐free except for a slightly limited range of motion of the left shoulder: 130° in flexion, 140° in abduction, 60° in external rotation at the side, and hand to the L3 level in internal rotation. Clinical scores improved, with a Constant–Murley score of 74, UCLA score of 29, and pain NRS of 1. No postoperative complications or adverse events were observed. The clinical course of this case is summarized in Table 1.

**Figure 6:**
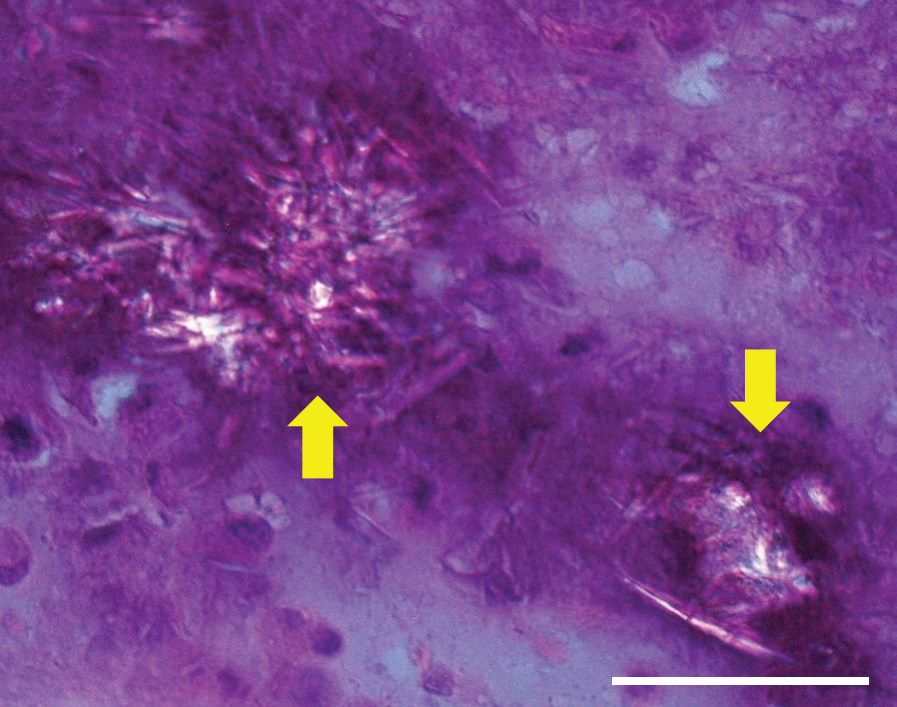
Photomicrograph of the lesion showing abundant angular and elongated crystalline deposits (arrows) (hematoxylin and eosin stain; scale bar, 50 *μ*m).

**Figure 7:**
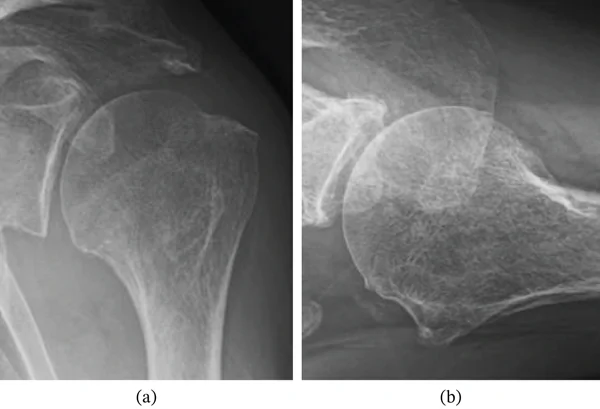
Postoperative radiograph of glenohumeral joint. (a) Anteroposterior view. (b) Axillary view.

**Figure 8:**
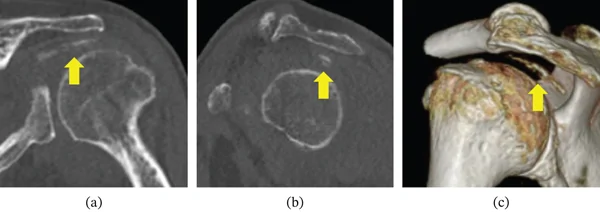
Postoperative computed tomography on the second day after surgery revealed residual calcific lesion (arrow). (a) Coronal image. (b) Sagittal image. (c) Three‐dimensional images.

**Figure 9:**
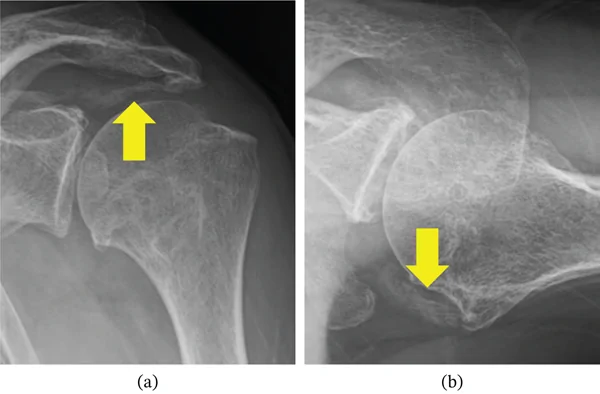
Postoperative radiograph at 2 years postoperatively showed slight enlargement of the residual calcific lesion. (a) Anteroposterior view. (b) Axillary view.

**Table 1:** Timeline of the clinical course of the patient.

Time point	Clinical events	Findings/assessments	Interventions
> 10 years before presentation	Onset of shoulder pain	—	—
2 years before presentation	Gradual worsening of symptoms	—	—
0 months (initial presentation)	Pain, swelling, limited ROM	ROM: flexion 60°, ER 30°, IR buttock; NRS 6; Constant 17; UCLA 10	—
	Preoperative evaluation	X‐ray/CT: calcified mass (41 × 24 mm); MRI: intra‐articular lesion; blood tests: normal	—
5 months after presentation	Surgery	Intra‐articular mass, partial supraspinatus tear	Arthroscopy‐assisted open resection of the mass
2 days postoperatively	—	CT: residual calcific deposits in supraspinatus tendon	Start rehabilitation
2 months postoperatively	—	Gradual pain relief and ROM improvement	Continued rehabilitation
24 months postoperatively	Final follow‐up	ROM: flexion 130°, ER 60°, IR L3; NRS 1; Constant 74; UCLA 29; slight enlargement of residual lesion	Observation

Abbreviations: Constant, Constant–Murley score; CT, computed tomography; ER, external rotation; IR, internal rotation; MRI, magnetic resonance imaging; NRS, numerical rating scale of pain; ROM, range of motion; UCLA, The University of California—Los Angeles shoulder score.

## 3. Discussion

Tophaceous pseudogout is a rare mass‐forming manifestation of CPPD caused by localized accumulation of CPP crystals. It is more commonly observed in the temporomandibular joint, knee joints, and digits [1, 2, 4, 5], and may occasionally cause bone destruction [6]. Reports of pseudogout with tumor‐like lesions in the glenohumeral joint are rare, with only one report found. Mizutani et al. [3] reported two shoulder lesions associated with bone erosion and destruction, whereas the present case showed no joint destruction. To our knowledge, this is the first report detailing the treatment and postoperative course of tophaceous pseudogout in the glenohumeral joint.

Preoperative diagnosis of tumoral CPPD remains challenging [7]. The differential diagnosis of a periarticular calcified mass includes crystal deposition diseases, tumoral calcinosis, synovial chondromatosis, and calcified or ossified soft‐tissue neoplasms. In the shoulder, hydroxyapatite deposition disease is an important differential diagnosis because it commonly produces amorphous calcific deposits within the rotator cuff tendons or periarticular soft tissues [8, 9]. In contrast, CPPD more typically involves articular structures such as cartilage, synovium, and the joint capsule, although tumoral CPPD may rarely form a mass‐like lesion. Tumoral calcinosis can also present as a periarticular calcified mass, but it typically demonstrates a multilobulated or cystic appearance, sometimes with fluid‐calcium levels on CT, and is often associated with abnormalities of phosphate metabolism [10]. Although both tumoral calcinosis and tumoral CPPD may show a chalky appearance macroscopically, the predominantly intra‐articular location and absence of the characteristic multilobulated or cystic appearance and fluid‐calcium levels favored tumoral CPPD in the present case. Histopathologically, the abundant angular and elongated crystalline deposits were morphologically consistent with CPPD; however, as discussed below, routine histopathological examination alone cannot definitively determine the composition of calcium‐containing deposits. Synovial chondromatosis generally presents with multiple rounded or nodular mineralized intra‐articular bodies rather than a diffuse calcified mass [11]. Calcified soft‐tissue neoplasms should also be considered, particularly when a nonmineralized soft‐tissue component, aggressive bone destruction, or infiltration of adjacent structures is present. In the present case, the intra‐articular location, absence of aggressive bone destruction or a substantial noncalcified soft‐tissue component, and the characteristic pathological findings ultimately favored tumoral CPPD. In retrospect, tumoral CPPD should have been included in the preoperative differential diagnosis because the lesion was predominantly calcified, located within the glenohumeral joint, and lacked aggressive imaging features. However, because tumoral CPPD involving the glenohumeral joint is extremely uncommon, this diagnosis was not sufficiently considered before surgery. This case therefore emphasizes the importance of including tumoral CPPD in the differential diagnosis of intra‐articular or periarticular calcified masses of the shoulder. Dual‐energy computed tomography (DECT) may provide complementary information in differentiating CPP from basic calcium phosphate deposits, although reliable discrimination remains limited in clinical practice [12, 13]. DECT was not performed in this case because tumoral CPPD was not suspected preoperatively.

Histopathological confirmation of CPPD crystals is important for establishing a definitive diagnosis [14]. Fine‐needle aspiration cytology has also been reported to be useful in the diagnosis of tophaceous pseudogout [15, 16]. However, cytological examination alone may not always provide a definitive diagnosis, and histopathological evaluation by open or excisional biopsy may be required [17]. In the present case, the diagnosis was based on the characteristic morphology of the crystalline deposits together with the overall clinicoradiologic–pathologic correlation. Polarized light microscopy could not be performed because of specimen exhaustion, and the limitations of the pathological assessment are discussed below.

Conservative treatment options include rest, cryotherapy, analgesics, and oral steroids, whereas surgical options range from tumor resection to total joint replacement [18, 19]. In this case, due to severe pain and limited range of motion, surgical resection was chosen despite the absence of joint destruction.

The need for preoperative biopsy should be determined based on the overall clinical and imaging findings [20, 21]. In the present case, preoperative biopsy was not performed because the lesion lacked aggressive imaging features, such as bone destruction or extension into the surrounding soft tissues. However, because a specific diagnosis could not be established preoperatively, biopsy might have been useful for diagnostic clarification and surgical planning.

Postoperative recurrence reports vary. Some reports indicate no recurrence within 2‐2.5 years postoperatively for tophaceous pseudogout in the fingers and knees [7, 22]. In contrast, recurrence has been reported in the fingers and elbows within 1‐12 years postoperatively [23, 24]. In this case, en bloc excision was performed under direct visualization, followed by arthroscopic excision of the residual calcific lesion. Surgical excision may be considered when tumoral CPPD causes persistent pain, mechanical symptoms, or marked functional limitation. In the present case, complete removal was difficult because calcific deposits extended into the torn rotator cuff. Although specific data on recurrence after intentionally incomplete excision are limited, residual CPP deposits may persist or enlarge over time, as observed in the present case. The residual calcific lesion showed mild enlargement during the 2‐year follow‐up period, although the patient remained largely asymptomatic. Previous reports have described both favorable outcomes without recurrence and delayed recurrence after surgical excision. Therefore, careful long‐term follow‐up may be warranted, particularly when complete excision cannot be achieved.

The primary limitation of this case is the absence of a preoperative biopsy, which might have facilitated earlier diagnosis and informed surgical planning. In addition, polarized light microscopy could not be performed due to specimen exhaustion, even though it is an important method for confirming the birefringent properties of CPP crystals. Routine histopathological examination alone has limitations in definitively identifying the composition of calcium‐containing deposits and cannot completely exclude other calcium phosphate deposits, particularly hydroxyapatite. Basic calcium phosphate crystals, including hydroxyapatite, are not reliably identified by conventional polarized light microscopy because of their small size and may require advanced analytical methods such as Raman spectroscopy [12]. Therefore, although the crystal morphology observed in this case was consistent with CPPD, the diagnosis was based on the characteristic histological appearance together with the intra‐articular distribution, imaging findings, and overall clinicoradiologic–pathologic correlation.

This case highlights the importance of considering CPPD in the differential diagnosis of tumor‐like calcified lesions of the shoulder to avoid misdiagnosis and unnecessary aggressive treatment.

## 4. Conclusion

Tophaceous pseudogout should be considered as a differential diagnosis when a calcified mass is located in a joint. Histopathological assessment, interpreted together with clinical and imaging findings, is important for establishing the diagnosis and distinguishing it from neoplastic and other calcium deposition disorders. Careful postoperative follow‐up is necessary because residual calcific deposits may persist or enlarge after incomplete excision.

## Funding

No funding was received for this manuscript.

## Conflicts of Interest

The authors declare no conflicts of interest.

## Data Availability

The data that support the findings of this study are available on request from the corresponding author. The data are not publicly available due to privacy or ethical restrictions.
